# Metabolic bioprofiling of different *Glycyrrhiza glabra* solvent fractions for the identification of anti-adenoviral compounds using LC-HRMS/MS and *in-vitro* cytopathic assay coupled with chemometry

**DOI:** 10.1186/s12906-023-04063-z

**Published:** 2023-07-21

**Authors:** Rahma SR. Mahrous, Hoda Fathy, Reham S. Ibrahim

**Affiliations:** Department of Pharmacognosy, Faculty of Pharmacy, 1 el-Khartoum square Azarita, Alexandria, 21521 Egypt

**Keywords:** *Glycyrrhiza glabra*, Human adenoviruse type-7, Multivariate analysis, Kaempferol-3-O-rutinoside, LC-HRMS/MS

## Abstract

Human adenovirus type-7 (HAdV-7) is a common pathogen that may cause significant morbidity as well as severe complications. Currently, there is no approved drug for the treatment of HAdV-7 infections and the contemporary strategy mainly rely on nonspecific antivirals. *Glycyrrhiza glabra*; (licorice), is a well-recognized edible plant used in food and beverages. The plant is renowned for its pharmacological and biological activities including antiviral activities against wide range of viruses. The following study reported for the first time the anti-adenoviral activity of licorice extract using *in-vitro* cytopathic inhibitory assay. Different solvent fractions showed promising activity with IC_50_ reaching 1.431 μg/ml. Certain fractions had selectivity index (SI) greater than 10 indicating their efficacy together with relatively low cytotoxic effect. Metabolic profiling using LC-HRMS/MS resulted in identification of 41 compounds from licorice fractions. Chemometric modeling using Orthogonal Projections to Latent Structures (OPLS) suggested the compounds; kaempferol-3-O-rutinoside, violanthin, rhamnoliquiritin, isoliquiritigenin isomer, licoagroside B and liquiritin apioside as potential markers against (HAdV-7). Finally, kaempferol-3-O-rutinoside was further confirmed via *in-vitro* adenovirus inhibitory assay to possess strong antiviral activity with IC_50_ and CC_50_ of 54.7 ± 1.93 μM and 655.7 ± 2.22 μM, respectively.

**Supplementary Information:**

The online version contains supplementary material available at 10.1186/s12906-023-04063-z.

## Introduction

Human adenoviruses (HAdV); with at least 51 serotypes; cause wide range of illnesses, including respiratory and gastrointestinal infections which are usually mild and heal on their own without the need for additional treatment [1]. However, Human adenovirus types 3 (HAdV-3) and 7 (HAdV-7) infect children worldwide, causing significant morbidity, severe problems, and long-term pulmonary damage [2]. Both types are the most common etiologic types of adenoviral pneumonia and lower respiratory diseases, especially in young children aging less than five [1, 2]. They were identified as the main epidemic types responsible for the severe (HAdV) pediatric infection outbreak in southern China in 2018 [3]. Adenovirus type-7 (HAdV-7) is more contagious than type 3 and causes a more severe infection in children as observed in many epidemiological studies [2, 4]. Moreover, It has been documented that some people, notably immunocompromised patients, have suffered severe and life-threatening adenoviral infections [5]. Currently, there are no specific drug or vaccine available for adenovirus infection. Thus, the search for drug candidates targeting these viruses is of massive significance [6].

The genus *Glycyrrhiza*, family Fabaceae comprises nearly 30 species of which, *Glycyrrhiza glabra* Linn. (licorice*)* is the most popular one [7]*.* It is native to Asia and the Mediterranean region and has been valued for its ethnopharmacological properties, particularly in Chinese traditional medicine, since ancient time [7, 8]*.*

Licorice roots contain a wide range of phytoconstituents such as triterpenes, saponins, flavonoids, coumarins, alkaloids, polysaccharides, and amino acids. The primary active ingredient of licorice is glycyrrhizin (triterpenoid saponin), responsible for its sweet taste. Almost 300 flavonoids of different classes are reported in licorice roots. They account for the root’s yellow color and include glycosides of the flavanone (liquirtigenin) and chalcones (isoliquirtigenin) as the major flavonoids [7–9]. Many pharmacological activities have been reported for licorice as, antioxidant, anti-inflammatory, antidiabetic, hepatoprotective, antitussive, anti-ulcerative, anticoagulant, anticancer, neuroprotective, antimicrobial, and antiviral effects [7, 8]. Licorice and its major compound, glycyrrhizin are widely used as flavoring agents. It is generally recognized as safe (GRAS) by the FDA, the Council of Europe, and the Joint FAO/WHO Expert Committee on Food Additives [7].

The plant is reputed for its antiviral activity against different DNA and RNA viruses such as herpes simplex, hepatitis viruses (types A, B & C), influenza, Japanese encephalitis, Varicella zoster, vesicular stomatitis, and human immunodeficiency virus (HIV). The primary active components with antiviral properties are the triterpenoids; glycyrrhizin and 18-β-glycyrrhetinic acid [10]. Glycyrrhizin, also known as glycyrrhizic acid was found to target the release step in which infectious anti-hepatitis C virus particles were infecting cells, played an important role in some viral infections such as human immunodeficiency virus, coxsackievirus B3, and had significant inhibiting effect of influenza virus at a concentration of 100 μg/ml [8, 10]. In addition, 18-β-glycyrrhetinic acid showed antiviral activity against retrovirus and human respiratory syncytial virus (HRSV). Studies showed that 18-β-glycyrrhetinic acid acts by inhibiting viruses’ replication and preventing their attachment to host cell [10]. It was reported that the flavonoids, isoliquiritigen, liquiritigenin, licochalcone A and glabridin had potent anti- hepatitis C activity in addition to the coumarins, glycycoumarin, glycyrin, glycyrol. These compounds are of common existence in different *Glycyrrhiza* species [11].

The current study was conducted to evaluate the anti-adenoviral action of licorice which hasn’t been studied before. Literature review revealed that licorice triterpenoids have potential antiviral properties [10, 12, 13]. We were interested in examining the antiviral activity of phenolic constituents of the plant against human adenovirus 7 (HAdV-7). As this class of compounds includes licorice flavonoids; the second major class known to exist in the plant following the triterpenoids [7]. In this context, the ethyl acetate fraction was extensively investigated using LC-HRMS/MS-based metabolomics coupled to chemometry and *in-vitro* cytopathic antiviral assay for the identification of potential anti-adenoviral compounds in *G. glabra* roots.

## Materials and methods

### Plant material and extract preparation

Licorice (*Glycyrrhiza glabra*) roots were collected from Alexandria, Egypt in December 2021. Permission was obtained for plant collection and the plant was collected according to the current national guidelines and was kindly identified by Professor Dr. Selim Zidan Heneidy, professor of Applied Ecology, Faculty of Science, Alexandria University. Voucher specimen (GG107) has been deposited in the herbarium of Pharmacognosy Department, Faculty of pharmacy, Alexandria University. The 70% ethanolic extract of powdered roots (85 g) was re-dissolved in 90% ethanol and fractionated successively with light petroleum followed by methylene chloride then ethyl acetate, and finally n-butanol to yield 7, 50, 10 and 6 g dry fractions, respectively.

### Fractionation and chromatographic separation of ethyl acetate fraction

In the present study, an evaluation of the ethyl acetate fraction of the root extract was conducted being rich in flavonoids and other phenolic compounds [14–16]. Consequently, the EtOAc fraction was selected for further purification and biological screening of its adenoviral inhibitory activity. The ethyl acetate fraction (10 g) was chromatographed on a silica gel gravity column. Gradient elution was performed using increased concentrations of methanol. fractions of similar TLC chromatographic pictures were pooled together, and six subfractions from 1 to 6 were obtained (Table 1).

**Table 1:** Subfractions of the EtOAc fraction of *G. glabra* root extract

Polarity (%methanol in methylene chloride)	Combined subfraction #	Weight (g)
0–5	**1**	1.2
5–6	**2**	1.74
7–11	**3**	0.95
12	**4**	1.35
13–15	**5**	0.45
16–100	**6**	1.91

### LC-HRMS/MS Data acquisition and sample analysis

Samples were analyzed in triplicates on an Orbitrap Fusion instrument (Thermo Fisher Scientific) controlled with Xcalibur version 2.1.1 (Thermo Fisher, San Jose, CA). Samples of the ethyl acetate fractions of *G. glabra* were loaded onto a C18 capillary column 100 Å pore (300 µm × 150 mm, 1.8 µm, Waters) and analyzed using mobile phase A (0.1% formic acid in water) and mobile phase B (0.1% formic acid in acetonitrile) at a rate of 5.0 μL/min. Elution was carried with a gradient consisting of 2 to 40% solvent B over 14 min, ramped to 95% B over 2 min, and then returned to 2% B (over 3 min and held for 17 min and the injector volume used was 8 μL. Compounds were eluted directly into the Orbitrap using HESI (heated electrospray ionization source). All data were acquired in positive ion mode. Full MS scans were acquired from *m/z* 200 to 2000. The voltage was 3500 V, and the ion transfer temperature was 300° C. The Orbitrap resolution was 60,000. Collision-induced dissociation (CID) was used for compound fragmentation with an isolation width of 3 m*/z* units.

### LC–MS Data mining and processing

LC–MS raw data files were imported to ProteoWizard 3.0 and converted to mzML format with MS Convert applications. This was followed by data processing and molecular feature detection using MZmine 2.0 (http://mzmine.sourceforge.net/) data analysis software. After deconvolution and alignment, a list of 300 resolved peaks including the identified compounds (41 compounds) was organized using (Microsoft Office Excel, 2013). Peak list of the identified compounds was imported into SIMCA-P version 14.0 software (Umetrics, Sweden) for further multivariate data analyses.

### *In-vitro* cytotoxic and antiviral activity against human adenovirus type 7

#### Cells and viruses

The Adenovirus type-7 (HAdV-7) and Hep-2 cells were provided by Nawah-Scientific, Egypt. Hep-2 cells were used for cytotoxic and antiviral studies. Hep-2 cells were grown in DMEM medium containing 10% fetal bovine serum and 0.1 percent antibiotic/antimycotic solution. The antibiotic and antimycotic solution, trypsine-EDTA, fetal bovine serum, and DMEM medium were all provided by Gibco BRL (Grand Island, NY, USA).

#### Samples

Samples of the ethyl acetate fractions were used at concentration range of 0.1–100 μg/mL. Samples were diluted with cell culture medium to the required concentrations using two-fold serial dilution and were used for both the cytotoxic and antiviral assays. Kaempferol-3-O-rutinoside was obtained from Sigma Chemical Co (USA). Stock solutions of the tested compound were prepared in 10% DMSO in double distilled water and further diluted to the working solutions with DMEM using two folds serial dilution.

#### Cytotoxicity assay

The cytotoxic effect of the fraction samples and top compound as revealed from multivariate model was determined prior to the antiviral assay using crystal violet method according to Schmidtke et al. [17]. In brief, cells were seeded at a density of 2 × 10^4^ cells/well in a 96-well culture plate. The next day, the culture medium containing serially diluted test samples of the ethyl acetate fractions and the tested compound was added to the cells and incubated at 37 °C in 5% CO_2_ for 48 h. Cells without samples' treatment served as cell controls. Following three times wash by PBS, the cell monolayers were fixed and stained with a 0.03% crystal violet solution in 2% ethanol and 3% formalin. After washing and drying, the optical density of individual wells was quantified spectrophotometrically at 540/630 nm and analyzed using automated ELISA techniques, DIAS (Dynex Immuno Assay System (DIAS, Guernsey, UK). The results of the 50% cytotoxic concentrations (CC_50_) were determined using GraphPad PRISM software (Graph-Pad Software, San Diego, USA) [18].

#### Antiviral activity

Antiviral activity of licorice fraction samples and top compound was evaluated using the cytopathic effect (CPE) inhibitory assay [17]. First, the infectivity of human Adenovirus type 7 was determined using the crystal violet method, which monitored cytopathic effect inhibitory assays and allowed the percentage of cell viability to be calculated using Hep-2 cells. The virus infective titer was determined and 0.1 mL of diluted virus suspension of (HAdV-7) containing CCID50 (50 percent cell culture infective dose) of virus stock was added to mammalian cells. This dose was selected to produce the desired CPEs after infection.

The cytopathic effect (CPE) inhibitory assay was performed in Hep-2 cells seeded into a 96-well culture plate at a density of 2 × 10^4^ cells/well one day before infection. The culture medium was removed the next day, and the cells were washed with phosphate-buffered saline. Then, cells were treated with mixture containing 0.1 mL of diluted virus suspension of HAdV-7 containing CCID50 and 0.01 mL of serially diluted samples of the ethyl acetate fractions and the tested compound and incubated at 37 °C in 5% CO_2_ for four days. The development of cytopathic effect was monitored by light microscopy. Following a PBS wash, the cell monolayers were fixed and stained with a 0.03% crystal violet solution in 2% ethanol and 3% formalin. The same procedure as described previously for the cytotoxicity assay was utilized and analyzed with DIAS system [17]. The percentage of antiviral activities of the ethyl acetate fractions were calculated according to Pauwels et al. [19] using the following equation:antiviral activity = [(mean optical density of cell controls − mean optical density of virus controls) / (optical density of test sample − mean optical density of virus controls)] × 100%where, virus controls, are the virus-infected cells without the samples tested. Cell controls are cells non-infected and not treated with the samples tested. Based on these results, the 50% CPE inhibitory dose (IC_50_) was calculated using GraphPad PRISM software (Graph-Pad Software, San Diego, USA).

### Statistical analysis

Chemometric orthogonal projection to latent structure (OPLS) model of the compiled LC–MS data matrices was conducted via SIMCA v 14 software (Umetrics, Sweden). Careful examination of the OPLS correlation coefficient plots enabled us to identify the metabolites strongly correlated to the investigated anti-adenoviral activity. Permutations plots were created to validate that the created models were not modelling the noise or over-fitted.

## Results and discussion

### Annotation of secondary metabolites of *G. glabra* ethyl acetate fractions by LC-HRMS/MS

Compound identification was based on accurate mass, retention time (Rt), and fragmentation pattern of compounds which were compared to literature data [9, 20–25] and data from multiple databases, including, DNP (Dictionary of Natural Products: www.dnp.chemnetbase.com), SUPERNATURAL II: (https://bioinf-applied.charite.de/supernatural_new/). Comparison with reference standards was also used whenever available. More than 100 compounds, including flavonoids, coumarins in addition to triterpene saponins, were resolved, of which 41 compounds were identified in licorice ethyl acetate fractions. The identified compounds arranged according to Rt are presented in (Table 2) with the diagnostic MS/MS fragmentation patterns and molecular formulae. The identified compounds belong to different phytochemical classes as shown.

**Table 2:** LC-HRMS/MS data of compounds identified in EtOAc fractions of *G. glabra* roots

**NO**	**R**_**t**_** (min)**	**Identification**	**Class**	**[M + H]**^**+**^	**Mass error** (**ppm)**	**Molecular formula**	**MS**^**n**^** ions**	**EtOAc Fraction No**^**b**^						**Ref**
								**1**	**2**	**3**	**4**	**5**	**6**	
1	10.269	Licoagroside B	Saccharolipids	433.1136	0.202	C18H24O12	163, 157, 127, 99	+	+	+	+	+	-	[21]
2	10.469	Kaempferol-3-O-rutinoside	Flavone O-glycosides	595.1663	-0.02	C27H30O15	286	+	+	+	+	+	+	[21]
3	10.563	Liquiritin apioside	Flavanone O-glycoside	551.1765	-0.01	C26H30O13	419, 257	+	-	+	-	+	+	[21, 25]
4	10.679	Liquiritin	Flavanone O-glycoside	441.1161^a^	-0.01	C21H22O9	280, 262	-	-	-	+	-	-	[9, 25]
5	10.918	Violanthin	Flavone C-glycoside	579.1707	-1.15	C27H30O14	561, 505, 489, 475, 459, 385, 355	+	+	+	+	+	+	[25]
6	11.304	Liquiritigenin 4'-[3-acetylapiosyl-(1–2)] glucoside	Flavanone O-glycoside	593.187	-0.15	C28H32O14	550, 533, 473, 461, 299, 257	+	+	+	+	+	+	[20]
7	11.377	Rhamnoliquiritin	Flavanone O-glycoside	565.1909	-2.12	C27H32O13	505, 445, 433, 419, 257	+	+	+	+	+	+	[20]
8	11.393	Esculin	Coumarin glycoside	341.1747	-1.79	C15H16O9	179, 163, 135, 124, 111	+	+	+	+	+	+	[21]
9	11.404	Licorice glycoside C2/A	Flavanone O-glycoside	727.2228	-1.35	C36H38O16	550, 532, 257, 137	-	-	-	+	-	-	[22, 25]
10	11.521	Licorice glycoside B/D1/D2	Flavanone O-glycoside	697.2121	-1.63	C35H36O15	651, 550, 532, 418, 257, 137	+	+	+	+	+	+	(22, 25)
11	11.667	3,3',4,4'-Tetrahydroxy-2 methoxychalcone	Chalcone	303.0862	-2.07	C16H14O6	288, 275, 152, 110	+	+	+	+	+	+	[22]
12	12.129	2',3',4',7-Tetrahydroxyisoflavan; (R)-form, 3',4'-dimethylether	Isoflavan	325.106^a^	2.671	C17H18O5	310, 295	+	+	+	+	+	+	[42]
13	12.314	Prunetin	Isoflavone	285.0757	-2.05	C16H12O5	269, 241, 137, 121	-	+	+	+	+	+	[43]
14	12.54	Kumatakenin	Flavone	315.086	-2.54	C17H14O6	300, 285, 287, 271, 149, 167	+	+	-	-	+	+	[32, 43]
15	12.57	Licochalcone B	Chalcone	287.0917	-0.92	C16H14O5	245, 193, 167, 147, 121	+	+	-	+	+	-	[30]
16	12.589	Licoagrochalcone D	Chalcone	355.1543	-0.78	C21H22O5	340, 337, 327, 234, 122	+	-	-	-	+	-	[22]
17	12.615	Liquirtigenin/ Isoliquiritigenin isomer	Chalcone	257.081	-1.64	C15H12O6	137, 121, 93	+	-	-	-	-	-	[21, 22]
18	12.664	Pinocembrin	Falvanone	257.0809	-1.78	C15H12O5	153, 131	-	+	+	+	+	+	[21, 25]
19	12.968	Echinatin	Chalcone	271.0967	-1.44	C16H14O4	256, 239, 151, 122	+	+	-	-	-	-	[21]
20	13.39	Echinatin isomer	Chalcone	271.0602	-1.65	C16H14O4	256, 227, 153, 135	+	+	+	+	+	-	[21]
21	13.449	Isoliquiritigenin isomer	Flavanone	257.081	-1.52	C15H12O7	137, 121, 93	+	-	-	+	+	-	[21, 22]
22	13.634	Formononetin	Isoflavone	269.0809	-2.01	C16H12O4	254, 237, 213, 107	+	-	-	+	+	+	[30]
23	13.718	Kanzonol U	2-arylbenzofuran flavonoid	309.1122	-1.52	C19H16O4	294, 172, 105	-	+	+	-	-	-	[8]
24	13.873	Hispaglabridin A	Pyranoisoflavan	393.2062	-0.92	C25H28O4	337, 191, 189	+	-	-	-	-	-	[21, 30]
25	14.069	Licoagrocarpin	Pterocarpan	339.1592	-1.19	C21H22O4	283, 255	+	-	-	-	-	-	[44]
26	14.237	3’-hydroxy-4’-O- methyl- glabridin	Pyranoisoflavan	355.1542	-1.13	C21H22O5	215, 189, 153, 147	+	-	-	-	-	-	[30]
27	14.406	Kanzonol X	Prenyl Isoflavan	395.2211	-2.92	C25H30O4	375, 349, 324, 215, 203	+	-	-	-	-	-	[21]
28	14.569	Erythrinin B	Prenyl Isoflavone	339.1229	-1.16	C20H18O5	321, 311, 295, 221, 119	+	+	-	+	+	-	[21]
29	14.69	Isoangustone A	Prenyl isoflavanones	423.1807	-0.11	C25H26O6	379, 367, 311, 176	+	+	+	+	+	-	[23]
30	14.983	Hispaglabridin B	Pyranoisoflavan	391.1907	-0.53	C25H26O4	335, 203, 189, 147	+	+	+	+	+	+	[21, 30]
31	15.279	Glycycoumarin	Coumarin	369.1336	0.628	C21H20O6	313, 285, 191,149	+	+	+	+	+	-	[21]
32	15.79	Glabrone	Pyranoisoflavone	337.107	-1.89	C20H16O5	319, 309, 295, 283, 137	-	-	-	+	-	+	[21, 25]
33	15.806	Glabridin	Pyranoisoflavan	325.1431	-2.87	C20H20O4	269, 203, 189, 123	+	-	-	-	-	-	[21, 25]
34	15.961	Glabrene	Pyranoisoflavene	323.1274	-2.77	C20H18O4	267, 213, 189, 123	+	-	-	-	-	-	[21, 25]
35	16.017	Kanzonol Y	Chalcone	411.2166	-1.4	C25H30O5	355, 235, 217, 189, 177, 161, 148	+	+	+	+	+	-	[9, 21]
36	16.051	Glabrol	Prenyl flavanone	393.2001	3.417	C25H28O4	351, 337, 205, 203	+	+	+	+	+	-	[29, 30]
37	16.225	Kanzonol Y isomer	Chalcone	411.2167	-1.18	C25H30O5	235, 217, 205, 177, 161, 135, 122	+	+	+	-	+	-	[21]
38	16.602	Licoisoflavone B	Pyranoisoflavone	353.1022	-1.05	C20H16O6	338, 335, 325, 311, 309, 201, 153	+	+	+	+	+	-	[22]
39	17.956	Licoisoflavone A	Prenyl isoflavone	355.1181	-0.27	C20H18O6	340, 337, 327, 311, 299, 203, 153	+	+	+	+	-	-	[22]
40	26.109	Kanzonol W; 4'-methylether	Pyranoisoflavone	351.123	-0.86	C21H18O5	336, 333, 323, 307	-	-	+	-	+	+	[45]
41	26.723	Glisoflavanone	Prenyl isoflavanones	425.196	-0.92	C25H28O6	369, 247, 231, 195, 111	-	-	-	-	+	-	[24]

^a^m/z values of [M + Na] + ions

^b^( +) meaning the presence of the metabolite in the investigated fraction, (-) meaning the absence of the metabolite in the investigated fraction

### Flavonoidal aglycones

Flavonoids are among the major bioactive constituents of licorice. These compounds undergo characteristic retro-Diels–Alder (rDA) fragmentation that was clearly observed in MS/MS fragments of the identified peaks. The loss of small molecules and/or radicals like H_2_O (18 Da), CH_3_ (15 Da), CO (28 Da) and CO_2_ (44 Da) from flavonoid skeleton was also noticed in MS^2^ spectra of flavonoids [26].

Typical flavonoids of licorice were observed as two base peaks (peaks **17** & **21**) at *m/z* 257.081, [M + H]^+^ appearing on different Rt where flavanone appears earlier than chalcones as previously reported [27, 28]. The fragmentation patterns of both compounds were very similar [29], where the main fragment ion, *m/z* 137 resulted from rDA cleavage (part A of rDA), in addition to fragment ion at *m/z* 121 (part B of rDA). The peaks were assigned as either liquirtigenin or isoliquirtigenin [22].

### Prenylated flavonoids

*G. glabra* specific chemical marker; glabridin (isoflavan) was identified at *m/z* of 325.1431 [M + H]^+^, (peak **33**) with fragment ion 189 (part A of rDA) predominating. Six prenylated isoflavans/ isoflavanone (peaks 24, 26, 27, 29, 30 & 41) were assigned as hispaglabridin A, 3’-hydroxy-4’-O- methylglabridin, kanzonol X, isoangustone A, hispaglabridin B and glisoflavanone, respectively. Five prenylated isoflavones, (peaks 28, 32, 38, 39 & 40) were identified namely, erythrinin B, glabrone, licoisoflavone B, licoisoflavone A, and kanzonol W; 4'-methylether, respectively based on comparison of their fragmentation pattern with those reported in literature [22, 25, 30]. Besides, one isoflavene; glabrene (peak 34) was detected at *m/z* 323.1274 [M + H]^+^ with fragments at *m/z* 123 and 189 produced through rDA reaction and fragment at 267 formed during the loss of C_4_H_8_ (-56 Da) from the protonated ion [M + H]^+^. One prenylated pterocarpan, (peak 25; licoagrocarpin) appeared as [M + H]^+^ ion at *m/z* 339.1592 and produced fragment ions at *m/z* 283 (loss of C_4_H_8_) and 255. Furthermore, prenylated flavanone as glabrol (peak 36) and chalcones as kanzonol Y and its isomers (peaks 35 & 37) were identified and confirmed from comparison of HRMS data with literature (Table 2).

To sum up, *Glychrriza* species is a well-known source of prenylated flavonoids [25, 31]. They share the dominance of fragment ion A of rDA fragmentation in their MS^2^ spectra. Besides the neutral loss of 42 Da (C_3_H_6_) and 56 Da (C_4_H_8_) observed indicating the degradation of pyran ring and/or prenyl chain [29]. This was concluded from fragments at 337 (hispaglabridin A), 335 (hispaglabridin B), 295 (glabrone), 269 (glabridin), 267 (glabrene), 355 (kanzonol Y), 351 (glabrol), 311 (licoisoflavone B), 299 (licoisoflavone A), and others (Table 2).

### Other flavonoids

Two isoflavonoids (peaks 13 & 22), and five chalcones (peaks 11, 15, 16, 19 & 20) were characterized and assigned as prunetin, formononetin, 3,3',4,4'-tetrahydroxy-2 methoxychalcone, licochalcone B, licoagrochalcone D, echinatin, and echinatin isomer, respectively. In addition, an isoflavan; 2',3',4',7-tetrahydroxyisoflavan-3',4'-dimethylether, one flavonoid, kumatakenin, and a flavanone; pinocembrin (peaks 12, 14 & 18) appeared as [M + Na]^+^ at *m/z* 325.106, and [M + H]^+^ at *m/z* 315.086, 257.0809, for the three compounds, sequentially. In addition to one pyrano-2-arylbenzofuran flavonoid, kanzonol U, peak **23** appeared as [M + H]^+^ at *m/z* 309.1122. The assignment was confirmed through comparison to previously reported HRMS data [8, 21, 32]. The MS^2^ spectra of these flavonoids showed the neutral loss of CH_3_, H_2_O, CO, and CO_2_ units (Table 2).

### Flavonoidal glycosides

Flavonoids O-glycosides were identified from the neutral loss of their corresponding sugar moieties. Most of these glycosides had the same aglycone; liquirtigenin which appeared as characteristic fragment at *m/z* 257 in the compounds liquiritin apioside, liquiritin, liquiritigenin 4'-[3-acetylapiosyl-(1–2)] glucoside, rhamnoliquiritin, licorice glycoside C2 and licorice glycoside D1 (peaks 3, 4, 6, 7, 9 & 10).

For example, the two compounds licorice glycoside D1 and licorice glycoside C2 had [M + H]^+^ ions at m/z 697.2121 and 727.2228. Their fragmentation produced very close fragment ions at *m/z* 550, 532, 257, and 137 that attributed to the loss of coumaroyl in licorice glucoside D1 and methoxy coumaroyl in licorice glycoside C2, followed by water loss, and the degradation of coumaroyl and saccharide moieties from both glycosides. Besides, fragment at *m/z* 137 indicates part A of rDA ion of liquirtigenin [20]. The flavanone glycoside: liquiritin appeared as [M + Na]^+^ at *m/z* 441.1161 and was unambiguously identified by comparing the MS/MS spectra with reference standard. Also, peak 3 with [M + H]^+^ at *m/z* 551.1765 was identified as liquiritin apioside and had fragment ions at *m/z* 419 and 257 attributed to the loss of apiose and disaccharide moieties, respectively.

Kaempferol-3-O-rutinoside (peak 2) appeared as [M + H]^+^ at *m/z* 595.1663 with fragment ion of *m/z* 286 indicating the loss of rutinose moiety. Besides, one C-glycosylated flavonoid; violanthin (peak 5) was identified and showed fragments at *m/z* 561 (loss of H_2_O) and at *m/z* 505, 489, 475, and 459 resulting from cross-ring cleavages of the glucose and rhamnose moieties that distinguished the C- glycoside fragmentation pattern from other O-glycosides detected in licorice [26]. This came in accordance with previously reported HRMS data of violanthin [25].

### Coumarins

Coumarins are among phenolic compounds known to exist in licorice [33]. The loss of CO_2_, CO and C_2_H_2_ is common fragmentation pattern of these compounds [34]. Two coumarins were identified in the investigated fractions of licorice (peaks **8** & **31**). They were confirmed based on comparison of their HRMS data with those previously reported in literature [9, 21]. Compound 8 had [M + H]^+^ ion at *m/z* 341.1747 and showed fragment ion at *m/z* 179 that was attributed to the loss of glucose moiety and this was followed by further elimination of CO_2_ yielding fragment ion at *m/z* 135. Thus, compound 8 was confirmed as esculin. Also, compound 31 had [M + H]^+^ ion of *m/z* 369.1336 with fragments at *m/z* 313 and 285 indicating the loss of prenyl chain then CO, sequentially, and was identified as glycycoumarin [35].

### Other compounds

One saccharolipid (peak 1) was identified, where it had [M + H]^+^ ion at *m/z* 433.1136. MS^2^ spectrum showed fragments at *m/z* 163, 157, 127, 99. Thus, it was deduced as licoagroside B based on comparing its HRMS data with those previously reported for *Glychrriza* species [21].

#### Cytotoxicity and anti-adenovirus activity of licorice ethyl acetate fractions on Hep-2 cells

**Fig. 1:**
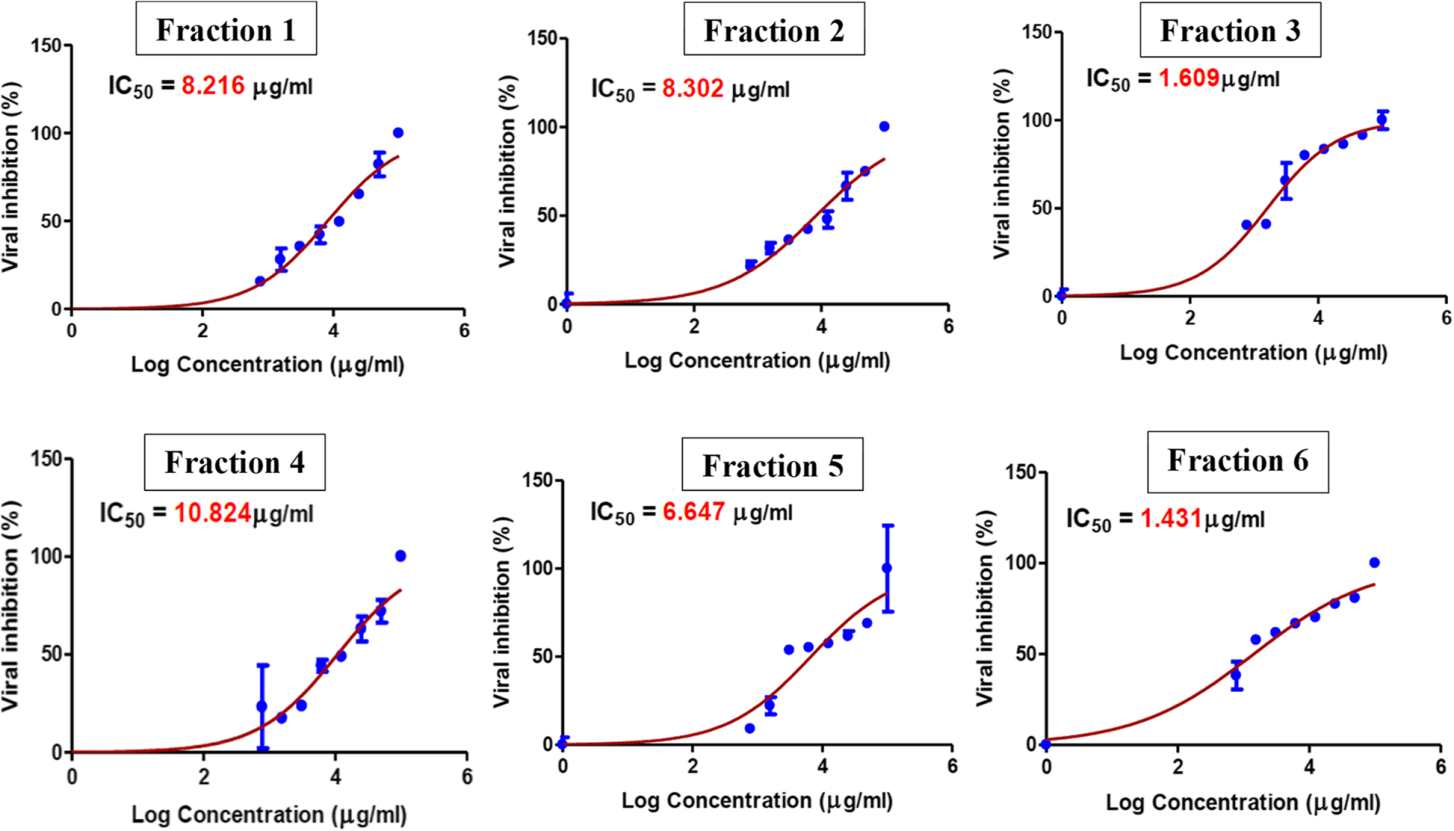
Dose response curve demonstrating 50% inhibitory concentration (IC_50_) on human AdV-7 virus of different licorice solvent fractions

**Fig. 2:**
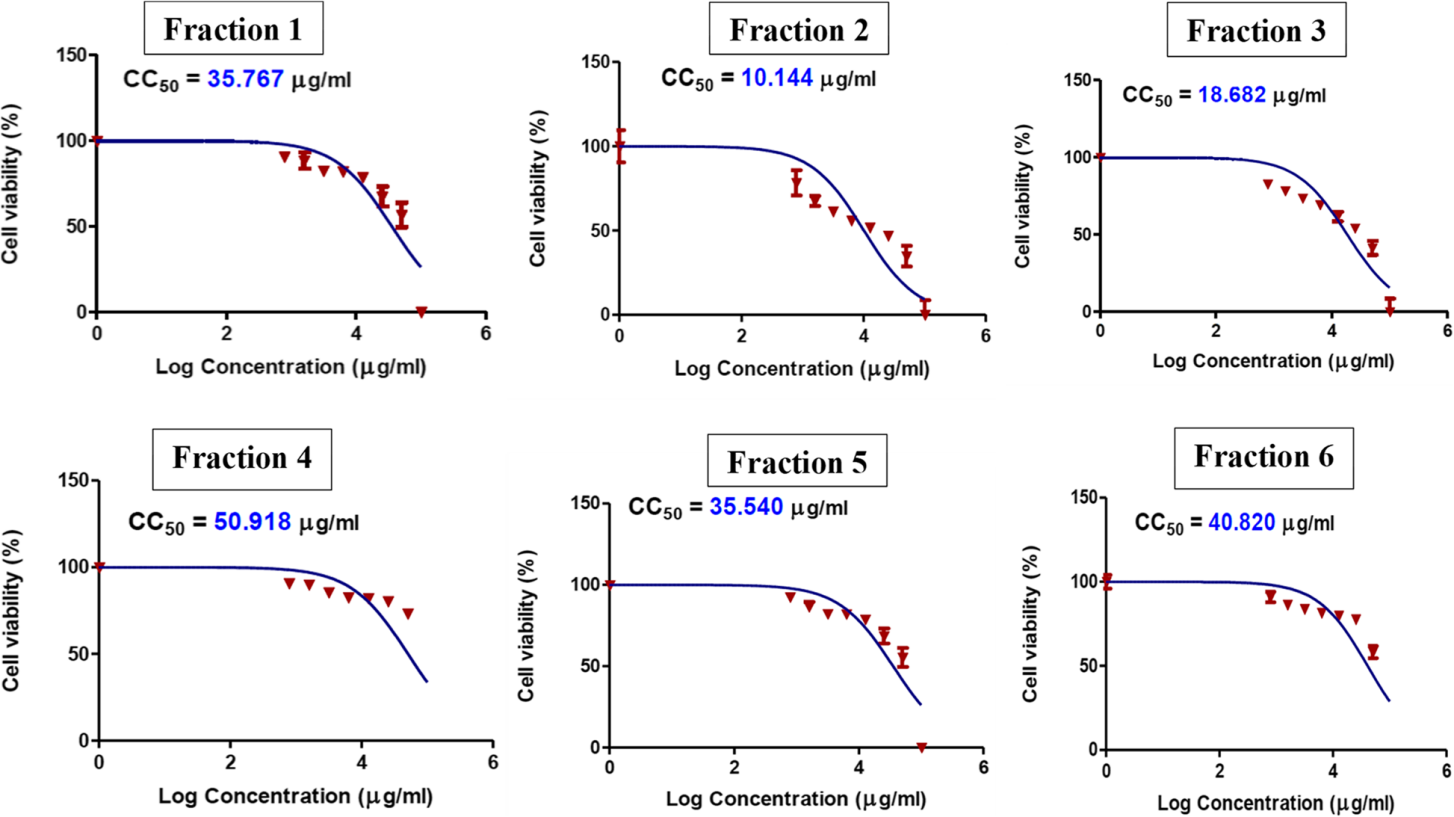
Dose response curve demonstrating 50% cytotoxic concentration (CC_50_) on normal Hep-2 cells of different licorice solvent fractions

The results of the *in-vitro* antiviral activity assay showed that all the tested ethyl acetate fractions of *G. glabra* possess strong inhibitory action on HAdV-7 with IC_50_ values on virally infected cells lower than 11 μg/mL indicating their potency (Table 3, Fig. 1). Fractions 3 and 6 were found to have the soundest antiviral activity with IC_50_ of 1.609 and 1.431 μg/mL, respectively. The cytotoxicity of the tested fractions was examined, to identify maximum non cytotoxic dose, where normal Hep-2 cells were still alive. The tested fractions had cytotoxic activity with CC_50_ values in the range of 10–50 μg/mL (Table 3, Fig. 2). Accordingly, selectivity index (SI) was calculated using the ratio of CC_50_ to IC_50_ [36]. Selectivity index (SI = cytotoxicity/bioactivity) appeared to be an indispensable parameter to evaluate during the exploring process of novel antiviral candidates rather than focusing only on pharmacological or toxicological parameters separately [37]. Among the tested fractions, fractions 3 and 6 had promising SI (Table 3), (SI ≥ 10) thus, could be assumed as bioactive and non-toxic fractions and are suggested for further investigations towards identifying lead compounds with potential anti-adenoviral activity.

**Table 3:** Cytotoxicity, anti-HAdV-7 activities and selectivity indices of licorice ethyl acetate fractions on Hep-2 cells*

Fraction #	CC_50_^a^(μg/mL)	IC_50_^b^(μg/mL)	SI^c^
1	35.767 ± 1.14	8.216 ± 1.62	4.353335
2	10.144 ± 1.78	8.302 ± 0.83	1.221874
3	18.682 ± 1.67	1.609 ± 1.59	11.61094
4	50.918 ± 2.19	10.824 ± 2	4.704176
5	35.54 ± 0.51	6.647 ± 2.05	5.346773
6	40.82 ± 1	1.431 ± 1.5	28.52551

^*^Results are expressed as mean ± SD of duplicate measurements

^a^50% cytotoxic concentration on normal Hep-2 cells

^b^50% inhibitory concentration on HAdV-7

^c^selectivity index

#### Correlation analysis to anti-adenoviral activity for unraveling bioactive phytoconstituents from the tested *G. glabra* fractions

**Fig. 3:**
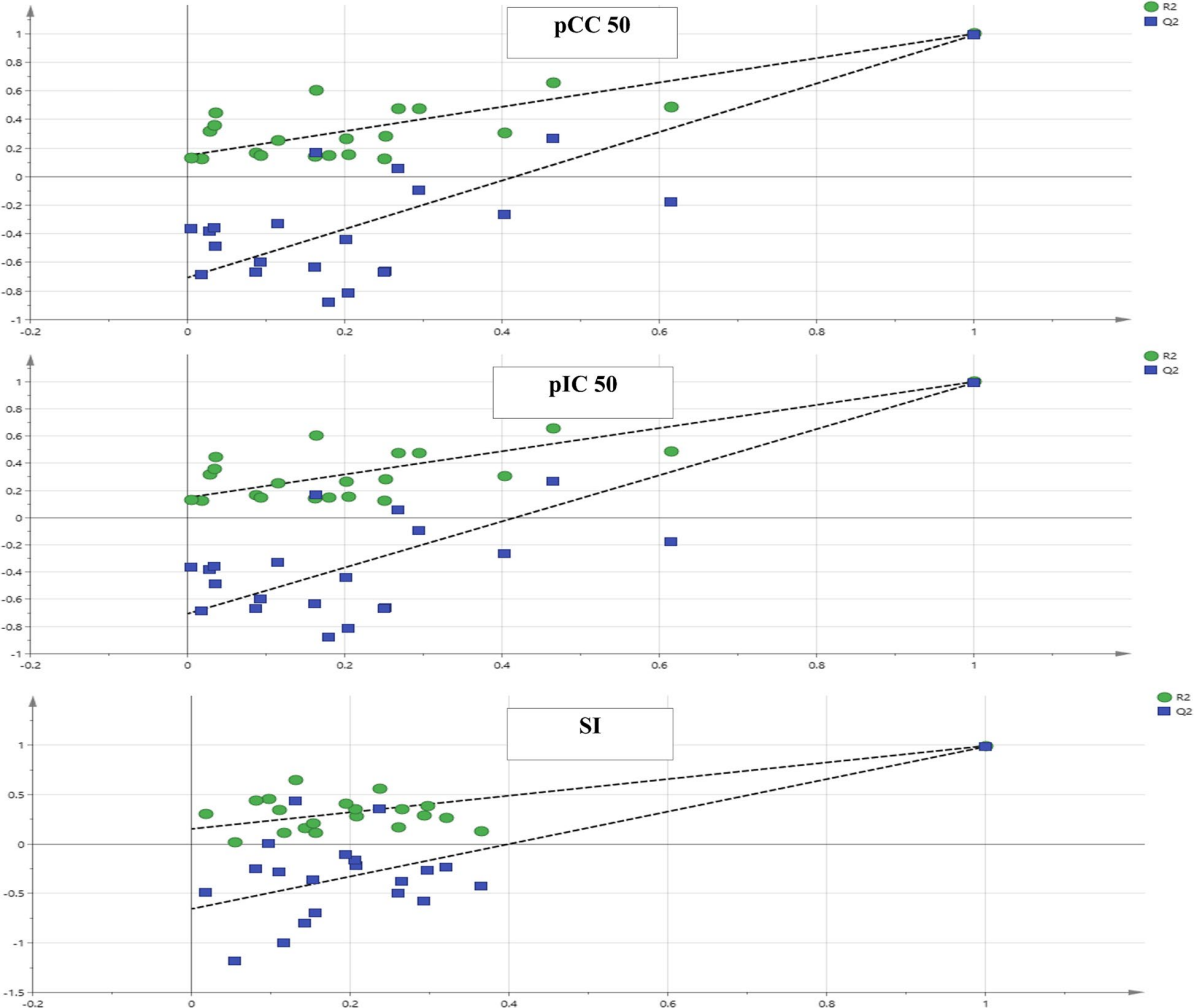
Permutation plots of OPLS model

OPLS model and its associated correlation coefficient analysis were executed for detection of putative markers having effective antiviral activity against HAdV-7 from the six licorice ethyl acetate fractions studied, as well as evaluating consequent classification of the fractions based on bioactivity. The model exhibited high reliability and prediction ability represented by high goodness of fitness (R^2^ = 0.995) and goodness of prediction (Q^2^ = 0.988). For validation of the current OPLS model; permutation plots for pCC_50_, pIC_50_ and SI (Fig. 3) using 20 permutations for each class were constructed. The blue regression line of Q^2^ points intersected with vertical axis below the zero, while the green R^2^ values to the left were lower to the original point to the right which strongly indicated the model validity.

**Fig. 4:**
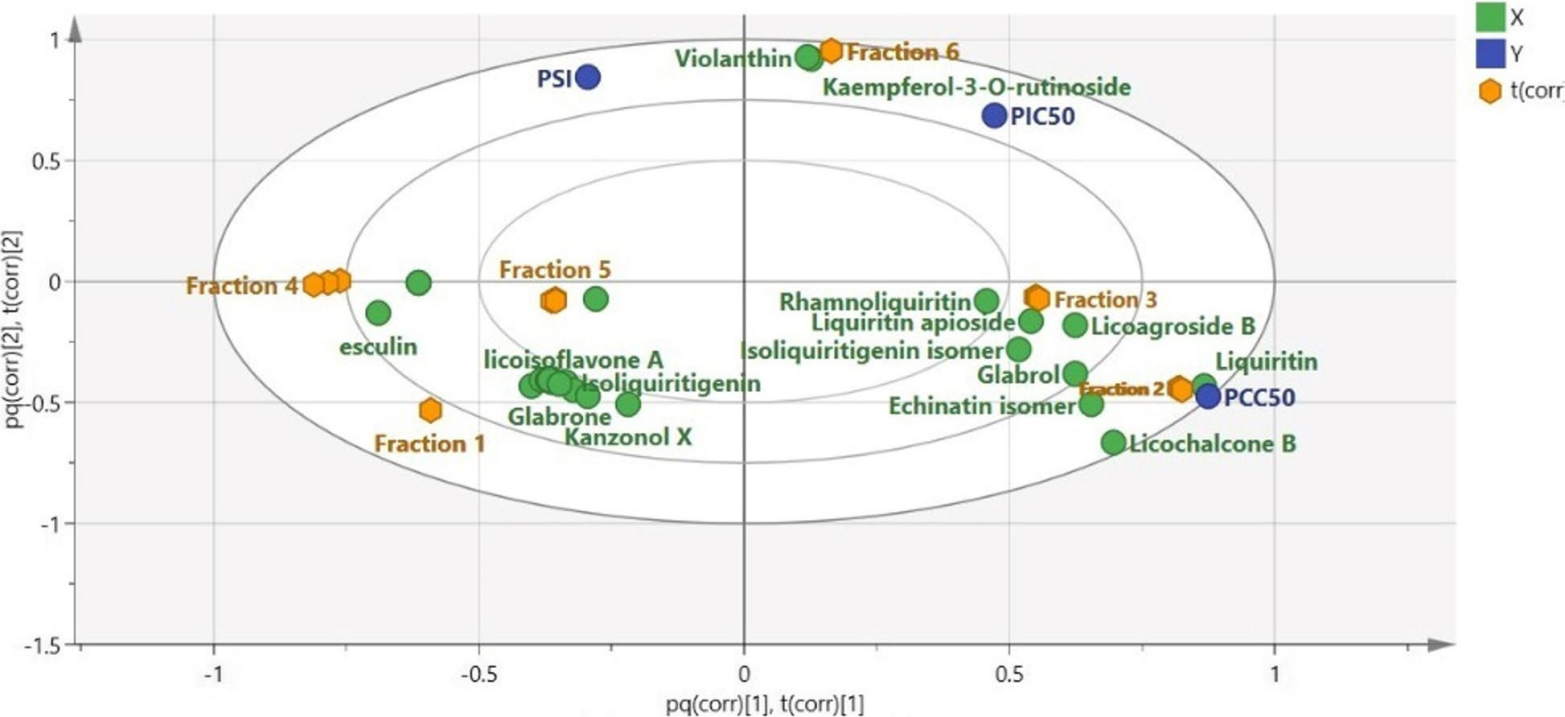
Orthogonal Projections to Latent Structures (OPLS) biplot of the tested fractions in correlation to the bioactive markers

**Fig. 5:**
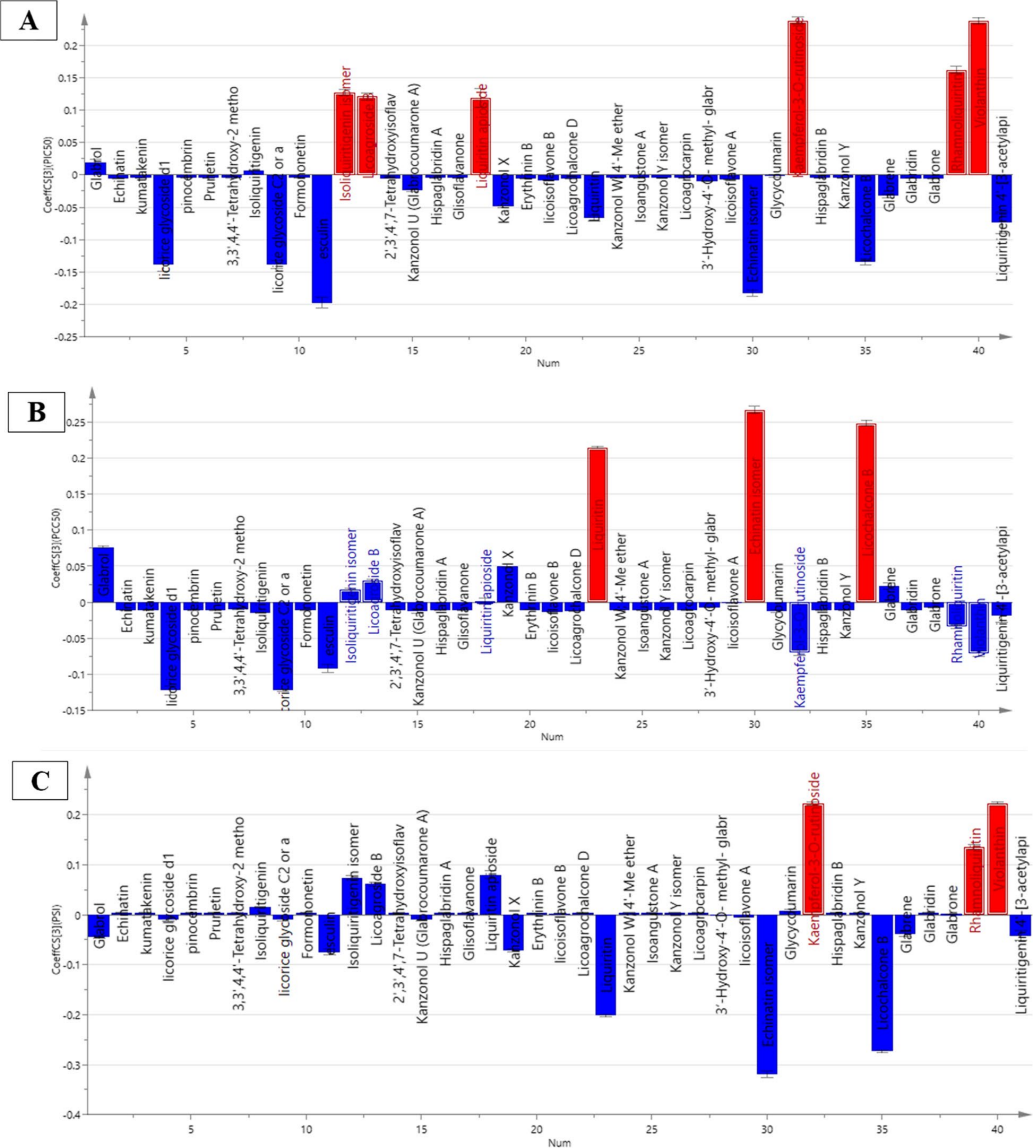
Coefficient plots of OPLS model in order to determine biomarkers responsible for the antiviral activity (PIC_50_) (A), cytotoxicity (PCC_50_) (B), and selectivity (PSI) (C)

The biplot of the constructed OPLS model (Fig. 4) revealed that fractions 2 and 3 exhibited spatial relation to cytotoxicity represented as pCC_50_, while fraction 6 was in proximity to pIC_50_ and SI indicating better antiviral activity on HAdV-7, respectively. Further, studying the coefficient plots (Fig. 4) portrayed that kaempferol-3-O-rutinoside, violanthin, rhamnoliquiritin, isoliquiritigenin isomer, licoagroside B and liquiritin apioside were shown to be the constituents possessing the highest positive correlation to HAdV-7 inhibitory activity (Fig. 5A). While echinatin isomer, licochalcone B and liquiritin were the major metabolites positively related to cytotoxic activity on normal cells (Fig. 5B). Finally, Fig. 5C indicated that kaempferol-3-O-rutinoside (Nicotiflorin) followed by violanthin then rhamnoliquiritin were the most potentially selective antivirals against HAdV-7. Antiviral activities of these flavonoids were reported against different DNA and RNA viruses [38]. For instance, kaempferol-3-O-rutinoside has shown activity against Herpes simplex virus 1 and 2 infection in *in-vitro* testing, and against coronavirus in a molecular docking study [38–40], while violanthin has been suggested by a docking study to have antiviral activity against coronavirus (SARS-CoV-2) [41]. Investigating the base peak chromatogram of the most active EtOAc fraction (fraction 6) (Figure S1) showed that it contains the three compounds suggested to have selective antiviral effect against HAdV-7, while lacking compounds responsible for cytotoxic activity on normal cells tested *in-vitro*. A list of the compounds identified in this fraction is given in supplementary file (Table S1).

#### *In-vitro* anti-adenoviral activity of kaempferol-3-O-rutinoside

**Fig. 6:**
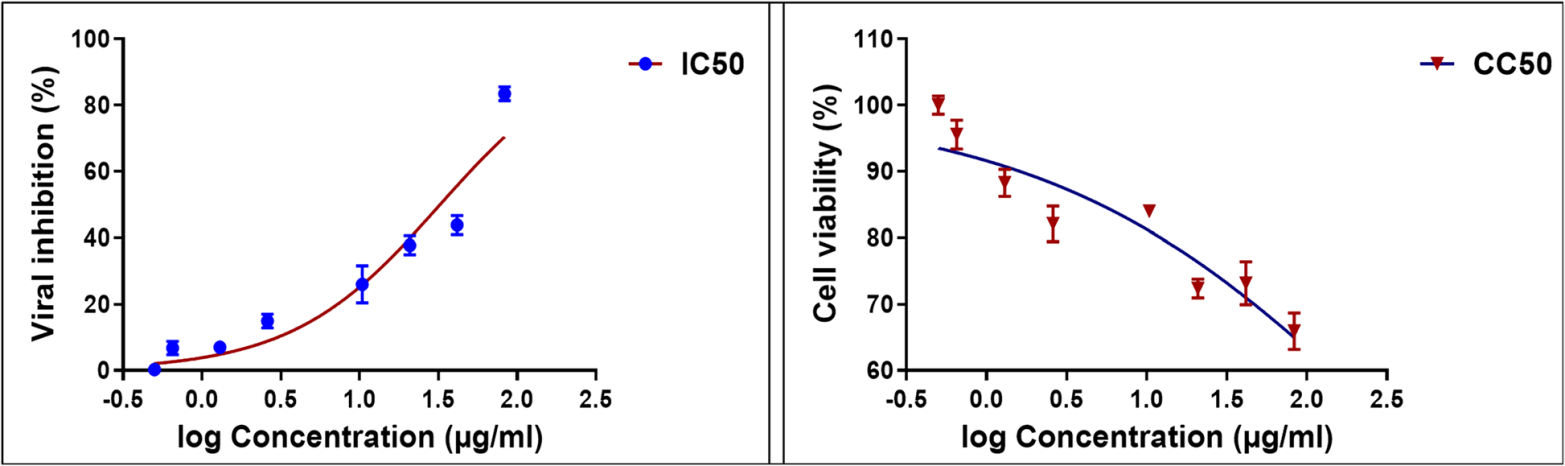
Dose response curve demonstrating 50% inhibitory concentration (IC_50_) on human AdV-7 virus (to the left) and 50% cytotoxic concentration (CC_50_) on normal Hep-2 cells (to the right) of kaempferol-3-O-rutinoside

The compound with the highest predicted anti-adenoviral activity based on correlation plot of selectivity index (SI); kaempferol-3-O-rutinoside was further evaluated *in-vitro* for its inhibitory action on HAdV-7 using the same cytopathic effect inhibitory assay. The results (Fig. 6) showed that the compound is a potent inhibitor of HAdV-7 with SI equal to 12 while having low toxic effect on the normal cell line tested (CC_50_ of 655.7 ± 2.22 μM) while maintaining high efficiency on virally infected Hep-2 cells (IC_50_ of 54.7 ± 1.93 μM).

## Conclusion

The study in hand provides a comparative evaluation of the metabolome of licorice different solvent fractions applying LC-HRMS/MS coupled with multivariate analysis. All studied licorice solvent fractions were tested for antiviral activity against human adenovirus (HAdV-7), and they all exhibited dose dependent inhibitory activity with variable degrees of safety, efficacy, and selectivity. Amongst the six studied solvent fractions, fractions 3 and 6 showed very strong activity against HAdV-7 with (SI > 11). Further, OPLS models and its accompanying correlation analysis were implemented for detection of putative phytoconstituents having effective, safe, and selective antiviral activity. Kaempferol-3-O-rutinoside was unraveled as an effective anti- adenovirus compound having great impact on safe and effective action of licorice subfractions. *In-vitro* testing confirmed its potency against HAdV-7.

## Supplementary Information

**Additional file 1: Fig. S1.** LC/HRMS base peak chromatogram of the most active EtOAc fraction (Fr 6) of *G. glabra* roots. **Table S1.** List of compounds identified in the most active EtOAc fraction (Fr 6) of *G. glabra* roots.

## Data Availability

The datasets used and/or analysed during the current study are available from the corresponding author on reasonable request.

## Abbreviations

CPE: Cytopathic effect
DNP: Dictionary of Natural Products
HAdV-3: Human adenovirus type-3
HAdV-7: Human adenovirus type-7
OPLS: Orthogonal Projections to Latent Structures
rDA: Retro-Diels–Alder
SI: Selectivity index
