# Supplementary material for: Metabolic bioprofiling of different Glycyrrhiza glabra solvent fractions for the identification of anti-adenoviral compounds using LC-HRMS/MS and in-vitro cytopathic assay coupled with chemometry
Source: BMC Complement Med Ther. 2023 Jul 21;23:259. doi: 10.1186/s12906-023-04063-z (PMC10362705; doi:10.1186/s12906-023-04063-z)
Supplement: Supplementary file 1 — Additional file 1: Fig. S1. LC/HRMS base peak chromatogram of the most active EtOAc fraction (Fr 6) of G. glabra roots. Table S1. List of compounds identified in the most active EtOAc fraction (Fr 6) of G. glabra roots. [file 12906_2023_4063_MOESM1_ESM.doc]

**Metabolic bioprofiling of different *Glycyrrhiza glabra* solvent fractions for the identification of anti-adenoviral compounds using** **LC-HRMS/MS and *in-vitro* cytopathic assay coupled with chemometry**

**Rahma SR. Mahrous ^1^, Hoda Fathy ^1^**^*^**, Reham S. Ibrahim ^1^**

^1^ Department of Pharmacognosy, Faculty of Pharmacy, Alexandria, Egypt

*** Correspondence:**Hoda Fathy*

hodasherif@hotmail.com, hoda.sherif@alexu.edu.eg

Address: 1 el-Khartoum square – Azarita, Tel: +2 01001223411, Fax: 4873273-4871668, Postal code: 21521

**Supplementary files**

| 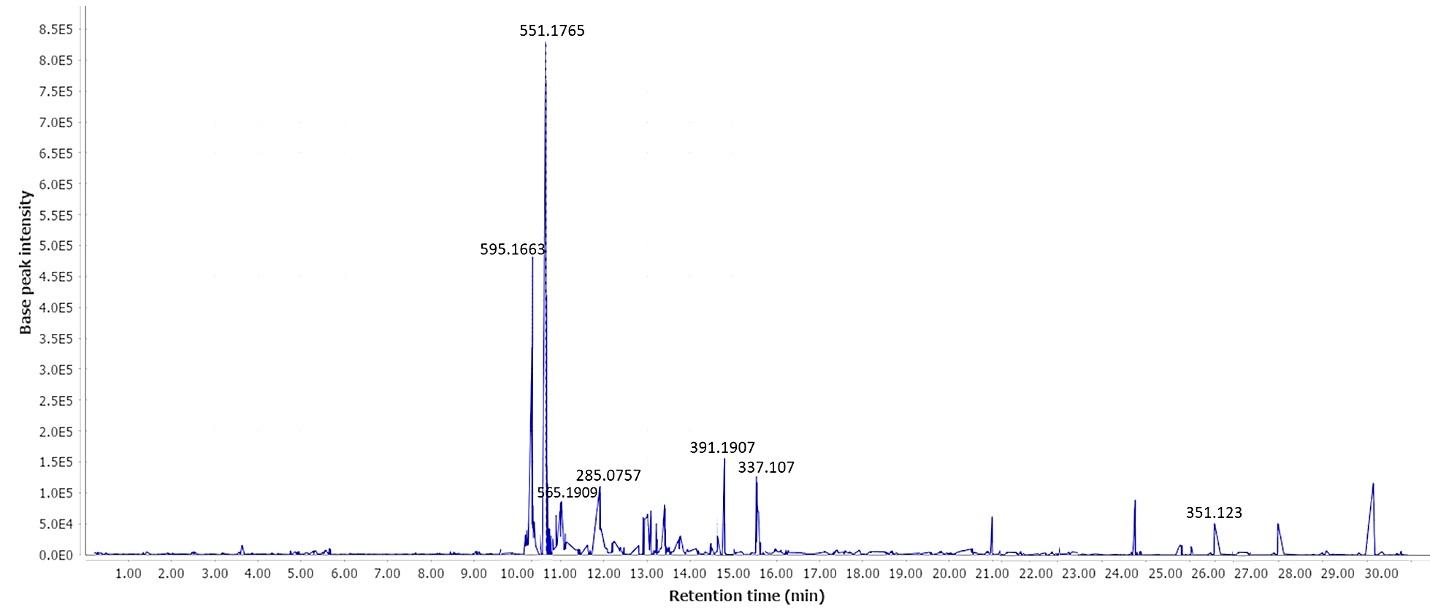 |
| --- |
| **Figure S1. LC/HRMS base peak chromatogram of the most active EtOAc fraction (Fr 6) of *G. glabra* roots** |

**Table S1: List of compounds identified in the most active EtOAc fraction (Fr 6) of *G. glabra* roots**

| **NO** | **Rt (min)** | **Identification*** | **Class** | **[M +H ]^+^** |
| --- | --- | --- | --- | --- |
| 1 | 10.469 | Kaempferol-3-O-rutinoside | Flavone O-glycoside | 595.166 |
| 2 | 10.563 | Liquiritin apioside | Flavanone O-glycoside | 551.177 |
| 3 | 10.918 | Violanthin | Flavone C-glycoside | 579.171 |
| 4 | 11.304 | Liquiritigenin 4'-[3-acetylapiosyl-(1-2)] glucoside | Flavanone O-glycoside | 593.187 |
| 5 | 11.377 | Rhamnoliquiritin | Flavanone O-glycoside | 565.191 |
| 6 | 11.393 | Esculin | Coumarin glycoside | 341.175 |
| 7 | 11.521 | Licorice glycoside B/D1/D2 | Flavanone O-glycoside | 697.212 |
| 8 | 11.667 | 3,3',4,4'-Tetrahydroxy-2 methoxychalcone | Chalcone | 303.086 |
| 9 | 12.129 | 2',3',4',7-Tetrahydroxyisoflavan; (R)-form, 3',4'-dimethylether | Isoflavan | 325.106* |
| 10 | 12.314 | Prunetin | Isoflavone | 285.076 |
| 11 | 12.54 | Kumatakenin | Flavone | 315.086 |
| 12 | 12.664 | Pinocembrin | Falvanone | 257.081 |
| 13 | 13.634 | Formononetin | Isoflavone | 269.081 |
| 14 | 14.983 | Hispaglabridin B | Pyranoisoflavan | 391.191 |
| 15 | 15.79 | Glabrone | Pyranoisoflavone | 337.107 |
| 16 | 26.109 | Kanzonol W; 4'-methylether | Pyranoisoflavone | 351.123 |

* m/z values of [M +Na ]+ ions
